# High serum Androgen and Insulin concentrations increase the tendency of Endometrial Carcinoma

**DOI:** 10.7150/jca.46391

**Published:** 2020-07-25

**Authors:** Fei Teng, Xiaotong Ma, Xiaopei Yu, Ye Yan, Jing Zhao, Jinping Gao, Chao Gao, Yingmei Wang, Wenyan Tian, Fengxia Xue

**Affiliations:** Department of Gynecology and Obstetrics, Tianjin Medical University General Hospital, Tianjin, China.

**Keywords:** endometrial carcinoma (EC), testosterone, androstenedione, insulin, cancer risk

## Abstract

**Purpose:** The objective of the study was to evaluate the important role played by androgen and insulin in the development of endometrial carcinoma (EC), and their combined effect on EC risk.

**Methods:** We enrolled 510 type I EC patients and 510 age-, time-, and nationality-matched subjects into this study. Metabolic and hormonal parameters of enrolled subjects were examined. Univariate and multivariate logistic regression analyses for EC and control subjects were performed. Type I EC risk was evaluated with respect to testosterone, androstenedione, and insulin levels based on odds ratios (ORs) using stratified data.

**Results:** EC risk was positively associated with C-peptide, estrone, androgen (including testosterone and androstenedione) and insulin levels, BMI, WHR, family history of cancer, nulliparity, irregular menstruation, diabetes, and hypertension. In multivariate logistic regression models, high C-peptide and testosterone levels, diabetes, and hypertension were independent risk factors after adjustment for BMI, WHR, family history of cancer, high serum insulin, and estrone levels. Increased serum total testosterone and insulin levels were positively correlated with EC risk in total, premenopausal, and postmenopausal women. Androstenedione was correlated with EC in total and postmenopausal, but not in premenopausal subjects. Compared with higher testosterone and insulin, odds ratios (ORs) for higher testosterone with lower insulin and lower testosterone with higher insulin were decreased in total, premenopausal, and postmenopausal women. Similarly, compared to both higher FAI and insulin, ORs for higher FAI with lower insulin and lower FAI with higher insulin were decreased in all three groups. Coordinately, ORs for higher androstenedione with lower insulin and lower androstenedione with higher insulin were decreased in total and postmenopausal, but not premenopausal subjects.

**Conclusions:** These findings suggested that androgen and insulin were risk factors of type I EC, and relatively high levels of both testosterone and insulin synergistically affected EC risk.

## Introduction

Endometrial carcinoma (EC) is one of the most common malignant tumors of the female reproductive system and accordingly is a severe threat to female health. Its prevalence has increased yearly in the United States, and it is estimated that by the end of 2019, new cases of EC will reach 61,880 1. Similar trends have been observed in China 2.

In the past 30 years, EC has been broadly classified into two subtypes on the basis of histological characteristics, hormone receptor expression, and grade. Type II EC are described as non-endometrioid, high grade, aneuploid, hormone-receptor negative tumors that are associated with a higher risk of metastasis and a poor prognosis. In contrast, the most common type I EC are thought to represent estrogen driven mostly by low-grade, hormone-receptor-positive, endometrioid tumors strongly associated with obesity and other components of the metabolic syndrome. It is generally acknowledged that the main internal secretion factor leading to type I EC is estrogen; the over-stimulation of endogenous and/or exogenous estrogen facilitates the abnormal proliferation of the endometrium 3. Estrogen mediates endometrial cell growth, proliferation, apoptosis inhibition, and angiogenesis *in vitro* and *in vivo* by activating estrogen receptors (ERs) and downstream signaling pathways 4. Progesterone counteracts the effects of estrogen (i) by inhibiting ERα expression, (ii) by preventing stromal production of growth factors and subsequent transcription factor activation 5, 6, and (iii) by inducing apoptosis in endometrial epithelial cells via progesterone receptor (PR) action in stromal cells 7. Therefore, the deficiency of endogenous progesterone levels and diminished length of lifetime progesterone exposure are associated with the development of endometrial hyperplasia and an increased risk of type I EC 8.

It has also been reported that the morbidity of type I EC is associated with diabetes, hypertension, obesity, and polycystic ovary syndrome (PCOS), and these diseases (or risk factors) all are related to metabolic syndrome, which includes insulin resistance as a pathophysiological basis. Under conditions of insulin resistance 9, target organs tend to be insensitive to insulin, leading to increased levels of blood glucose, which makes β cells constantly secrete insulin and C-peptide, and triggering the rise of plasma insulin and C-peptide levels. Although insulin and C-peptide are secreted in equimolar amounts, C-peptide has a longer half-life than insulin and its circulating levels are subject to less fluctuation. Thus, C-peptide is considered to be an indirect indicator of insulin secretion. Hyperinsulinism plays an important role in carcinogenesis as it potentiates mitotic activity in the glands and stroma 10, 11. Moreover, insulin binding sites are also found in the endometrial stroma of women with EC 12. Therefore, excessive insulin signaling can result in endometrial changes, similar to the effects of unopposed estrogen. In addition, a higher level of insulin can facilitate the synthesis of androgen 13, which can be converted into estrogen by peripheral conversion under the action of aromatizing enzymes from the liver and fat, causing increased estrogen levels and thus an increased morbidity of EC.

Although we currently understand some aspects of the pathogenesis and etiology of type I EC, there are still many unresolved issues regarding the whole EC disease and reproductive health system. For example, for a long time, researchers have consistently focused on the roles of estrogen and progestational hormone in the pathogenesis of type I EC 3, but have overlooked the possible influence of other hormones. Therefore, it is very important to clarify the high-risk internal secretions resulting in type I EC and even potential blood diagnostic indicators. A recent study has reported high androgen levels as a risk factor of EC 14. In fact, the prevalence of EC is higher in postmenopausal women than in women during reproductive years. However, we cannot ignore the irregular non-ovulatory menstrual cycles in perimenopausal periods also expected to increase the risk of EC. But it is difficult to define accurately whether women are experiencing perimenopause. Therefore, in the present study, we examined the roles of sex hormones and insulin in total, premenppausal, and postmenppausal women. We determined the correlations of androgen and insulin levels in serum with type I EC risk, and the combined effect of androgen and insulin on type I EC. These findings contribute to the diagnosis and pathogenesis of type I EC.

## Materials and Methods

### Patient samples and control subjects

A total of 510 patients with type I EC and 510 control patients at Tianjin Medical University General Hospital were included in the study and were recruited from August 19^th^, 2003 to November 4^th^, 2015. Inclusion criteria were the patients with the first diagnosis of histologically confirmed type I EC and without other malignancies, none of whom received hormone therapy, radiotherapy, or chemotherapy prior to surgery. Control subjects were selected among women who presented for routine examination in the general physical examination center. None had a history of cancer, and they were age-, time-, and nationality-matched to the patients. The exclusion criteria for all the subjects were as follows: thyroid diseases, hepatic dysfunction, renal inadequacy, non-classical congenital adrenal hyperplasia (NCAH), androgen-secreting tumors or use of medicines in the past 6 months that affect reproductive or metabolic functions. No history of oophorectomy.

Clinicopathological information was blindly and independently examined by two pathologists. Height, weight, waistline, abdominal perimeter, and hipline were measured immediately after serum collection for all patients and healthy subjects. Information on nulliparous, age at menopause, irregular menstruation, diabetes, hypertension, and family history of cancer was also collected from each subject. Using these parameter estimates, the waist-to-hip ratio [WHR, waistline (m)/hipline (m)], body mass index [BMI, weight (kg)/height^2^ (m^2^)], and homeostasis model assessment-insulin resistance [HOMA-IR; plasma glucose (mmol/L) × serum insulin (mIU/L)]/22.5] were calculated.

### Serum collection

One 5-mL blood specimen was collected early in the morning after 6 hours of fasting and prior to surgery (for patients) or on the day of regular examination (for healthy subjects). The samples were centrifuged (1000 × *g*, 10 min), and the serum was separated and stored at -80°C. Sex hormones (including total testosterone, androstenedione, estradiol and estrone) and insulin were examined according to the manufacturers' (see *Serum analysis* section for details) instructions.

### Serum analysis

The Estrone Radioimmunoassay Kit (Diagnostic Systems Laboratories, Inc., Webster, TX, USA) was used to quantify serum levels of estrone. Chemiluminescence reagents used to quantify serum levels of insulin, estradiol, total testosterone and sex hormone-binding globulin (SHBG) were obtained from Siemens Medical Solutions (Malvern, PA, USA). The Active Androstenedione Radio Immunity Assay kit (Beckman Coulter, Inc., Miami, FL, USA) was used for the androstenedione analysis. The electrochemiluminescence method (Roche Diagnostics GmbH, Mannheim, Germany) was used to assess the levels of C-peptide. The free androgen index (FAI) was calculated as:

FAI = [total testosterone (nmol/L)/SHBG (nmol/L)] × 100

where 1.00 nmol/L total testosterone = 28.84 ng/dL total testosterone. Experimental results are expressed as means ± SD of three independent experiments for each sample.

### Statistical analysis

Conditional logistic regression was used to analyze the risk factors for EC. Multiple comparisons were analyzed using Wilcoxon sign-rank tests or one-way ANOVA. A subsequent least significant difference (LSD) test was used if the variances were equal, and Tamhane's T2 test was used if the variances were unequal. The association between androgen levels and EC risk, and the association between androgen levels and clinicopathological characteristics were measured by unconditional logistic regression. Odds ratios (ORs) and 95% confidence intervals (CIs) were estimated for the associations between total testosterone, androstenedione, insulin, other potential risk factors, and EC. All statistical tests were two-sided. Analyses were performed using the SPSS 18.0 package.

## Results

### Basic characteristics of type I EC and control subjects

Here, only the type I EC was under investigation. The basic characteristics of the total, premenopausal, and postmenopausal subjects (EC cases and healthy controls), including marker concentrations in serum, are displayed in **Table 1.** Compared with the control subjects, the weight, waist circumference, abdominal circumference, hip circumference, BMI, WHR, and HOMA-IR were significantly higher in the total, premenopausal, and postmenopausal patients with EC than in controls (*P* < 0.05). Additionally, diabetes, hypertension, irregular menstruation, and family history of cancer were also more prevalent among the total, premenopausal, and postmenopausal patients with EC than in the control subjects (*P* < 0.05). The self-reported nulliparous history was more prevalent among the total and postmenopausal patients with EC than in the control subjects (*P* < 0.05). Conversely, there were no significant differences in age, height, and self-reported age at menopause between the total and postmenopausal groups (*P* > 0.05). Serum insulin, total testosterone, androstenedione, C-peptide and FAI levels were notably higher (*P* < 0.05) whereas SHBG level was significantly lower (*P* < 0.001) in the total, premenopausal, and postmenopausal EC subjects compared to the controls. Compared with the control subjects, the total and postmenopausal EC patients had significantly higher serum concentration of estrone (*P* < 0.001), but did not differ significantly in serum concentration of estradiol (*P* > 0.05). On the contrary, serum estradiol concentration was notably higher (*P* < 0.05) whereas estrone concentration was not notably higher (*P* > 0.05) in the premenopausal EC cases compared to the controls.

Additionally, of the 510 EC patients, 387 (75.88%) patients presented with International Federation of Gynecology and Obstetrics (FIGO) stage I, 32 (6.27%) cases with stage II, 73 (14.31%) ones with stage III, and 18 (3.54%) patients with stage IV disease. In terms of the histopathologic grade, there were 242 (47.45%) patients with G1, 212 (41.57%) ones with G2, and 56 (10.98%) cases with G3. There were 393 (77.06%) patients had < 1/2 myometrial invasion and 117 (22.94%) cases had ≥ 1/2 myometrial invasion.

### Serum androgen and insulin concentrations are independent risk factors for type I EC

Using a univariate logistic model, we detected positive associations between type I EC risk and serum C-peptide, estrone, insulin, and androgen (including testosterone and androstenedione) levels, BMI, WHR, family history of cancer, nulliparous, irregular menstruation, diabetes, and hypertension (*P* < 0.001; **Table 2**). Per multivariate logistic modeling, the testosterone concentration (OR, 1.791; 95% CI, 1.259-2.548; *P* = 0.001) retained a positive association with EC after adjustment for BMI, WHR, family history of cancer, high serum insulin and estrone levels, suggesting that the testosterone level represented an independent risk factor for type I EC. Furthermore, multivariate logistic regression analyses also revealed that C-peptide level, nulliparous, irregular menstruation, diabetes, and hypertension remained positively correlated with type I EC (**Table 2**).

### Relationship between testosterone, androstenedione, insulin and FAI levels with type I EC risk after stratification for BMI, WHR, diabetes, and hypertension

After EC risk factors, including BMI, WHR, diabetes, and hypertension, were stratified, the relationships between type I EC risk and total testosterone, androstenedione, insulin and FAI levels were analyzed in total women (**Table 3**). Testosterone was associated with increased EC risk upon stratification for BMI over 24 kg/m^2^ (OR, 1.885; 95% CI, 1.375-2.585; *P* < 0.001), WHR over 0.85 (OR, 2.058; 95% CI, 1.530-2.766; *P* < 0.001), and for women without diabetes (OR, 1.722; 95% CI, 1.308-2.266; *P* < 0.001), whereas there were no significant associations in subjects with BMI less than 24 kg/m^2^, WHR less than 0.85, and in women with diabetes (*P* > 0.05, Table 3). Insulin was found to increase EC risk in all stratified data (*P* < 0.05) except, in women with diabetes (*P* = 0.137, **Table 3**). Similarly, androstenedione and FAI were associated with increased EC risk in all of the risk factor stratifications (*P* < 0.05), except in women with WHR less than 0.85 (*P* = 0.054, **Table 3**).

### Higher serum androgen and insulin levels increase the risk for type I EC

To investigate whether insulin and androgen synergistically enhance patient risk for type I EC, we analyzed the odds ratios of total testosterone, androstenedione, FAI, and insulin for EC using a logistic regression model, according to stratified data. First, the total testosterone, androstenedione, and insulin concentrations were stratified into median values according to their distribution among EC patients and controls. The cut-off points for serum total testosterone, androstenedione, and insulin concentrations were 40.10, 39.02, and 41.00 ng/dL, 1.47, 1.75, and 1.39 ng/mL, and 8.82, 8.74, and 8.90 mIU/mL in total, premenopausal, and postmenopausal women, respectively. The FAI levels were divided into less than 5% and more than 5% among EC patients and controls.

Compared with the lower stratification groups, ORs for the higher stratifications in total, premenopausal, and postmenopausal women for total testosterone were 1.656 (95% CI, 1.293-2.121; *P* < 0.001), 1.851 (95% CI, 1.194-2.870; *P* = 0.006), and 1.520 (95% CI, 1.126-2.051; *P* = 0.006), respectively (**Table 4**). However, similar comparisons for androstenedione were not significant (*P* = 0.151; **Table 4**) in the premenopausal group. Additionally, an increased insulin level was positively correlated with EC risk in total (OR, 2.934; 95% CI, 2.275-3.783; *P* < 0.001), premenopausal (OR, 4.280, 95% CI, 2.695-6.796; *P* < 0.001), and postmenopausal women (OR, 2.414; 95% CI, 1.779-3.277; *P* < 0.001), respectively (**Table 4**).

To investigate whether high serum androgen and insulin levels were synergistic EC risk factors, we analyzed the ORs for testosterone, androstenedione, FAI, and insulin for EC using logistic regression models with stratified data. We found that compared with both higher testosterone and insulin, ORs for higher testosterone with lower insulin and lower testosterone with higher insulin were decreased in a stratified analysis of total, premenopausal, and postmenopausal women (**Table 4**). Coordinately, compared with both higher androstenedione and insulin, ORs for higher androstenedione with lower insulin and lower androstenedione with higher insulin were decreased in a stratification analysis of total and postmenopausal women (**Table 4**). Finally, compared to both higher FAI and insulin, ORs for higher FAI with lower insulin and lower FAI with higher insulin were decreased in all three groups (**Table 4**).

## Discussion

EC is the most common gynecological malignancy. About 14% of all cases are diagnosed in premenopausal women (a minority of which may have not yet completed childbearing), whereas the vast majority of EC occurs after menopause. Type I is the most commonly diagnosed form (about 80% of the cases) and is related to a prolonged estrogen exposure, as encountered in case of obesity, nulliparity, early menarche, late menopause (> 55 years), and polycystic ovarian syndrome (PCOS). However, in contrast to studies regarding estrogens, the possible impact of androgen and insulin actions on type I EC has largely remained unknown. Therefore, we discuss the effects of androgen and insulin in type I EC in this part.

### Occurrence of type I endometrial carcinoma is regulated by steroid hormones

Type I EC risk is thought to be associated with two major regulatory systems, i.e., sex hormonal mechanisms and the growth factor system. Large-scale case studies have supported these associations. More specifically, insulin resistance, steroid hormones, and inflammation are related to a high type I EC occurrence 15. The normal endometrium is regulated periodically by various hormonal signals from the ovaries (mainly estrogen, progesterone, and androgen), which act via their own hormone receptors to transmit signals 3. Currently, the long-term stimulation of estrogen without the antagonist progesterone is considered the main causal factor for EC. However, the theory of the progesterone-estrogen antagonism cannot account for all etiologies of type I EC.

In recent years, high androgen has been considered a new type I EC risk factor in women 16. As we known, only the free androgen fraction is able to diffuse into the cell and exert carcinogenetic effect by binding to the androgen receptor. Therefore, we use FAI to reflect the risk of EC caused by active androgens. Our results showed increased serum androgen levels in type I EC patients (**Table 1 & Table 4**), consistent with previous reports 17-19. However, these previous studies have not clarified the type I EC risk after adjusting for the estrogen level, as androgen could be converted to estrogen by aromatase and aldo-keto reductase 20 and further affects the estrogenic pathway via ERs. Additionally, it has been shown that androgen, after reaching a high level, can be converted to estrogen via aromatase 17, 18, thereby increasing the level of estrogen and thus the risk of EC. In general, research has firmly established that the metabolism of local androgen from type I EC tumor tissues may be more involved after transformation into estrogen. We analyzed the type I EC risk of androgen with adjustment for estrogenic factors to address this limitation. To be specific, our research indicates that androgen, especially testosterone, affects the occurrence of type I EC tumors as an independent risk factor (**Table 2**), and not only as the precursor of estrogen but also androgenic pathways. Researchers reported that androgen receptor (AR) expression within the healthy endometrial epithelium and hyperplastic tissue, but expression in EC. Furthermore, loss of androgen receptor (AR) in EC is associated with poor survival 21. These observations indirectly support the regulatory roles of androgen and ARs in the occurrence of EC.

### Occurrence of type I endometrial carcinoma is regulated by insulin levels

Insulin is considered as an important component of the growth factor system and high levels of insulin increased type I EC risk in our study, in accordance with previous results for other cancers 22, 23. Compared to androgen, the mechanism of insulin signaling in proliferative endometrial changes has been well-studied 24, 25. To be specific, insulin promotes EC progression directly via insulin signaling, and further activates various proliferation signals, including the PI3K/Akt and MAPK/ERK pathways 25. On the other hand, insulin can promote cancer indirectly via affecting sex hormone levels. Insulin can promote the generation of androgen by both ovarian and adrenal gland sources, stimulate the production of aromatase in endometrial stroma, and facilitate androgen conversion to estrogen 26. Moreover, the indirect cancer-promoting function of insulin also includes the inhibition of SHBG (which can tightly bind estradiol and testosterone) production in liver, elevating free sex hormones (both androgen and estrogen) in serum. In general, insulin can function indirectly via ERs and ER-mediated signaling.

As is common knowledge, the pathophysiological basis of metabolic syndromes, like obesity, hypertension, and diabetes, involves insulin resistance. Associations of morbidity of EC and these metabolic syndrome-related diseases (or risk factors) are well established. Generally, insulin, as a multi-functional protein hormone, not only widely regulates human metabolism, but also functions as a growth factor to stimulate the proliferation and differentiation of multiple cell types and to inhibit apoptosis. Therefore, hyperinsulinism or excess insulin can result in endometrial changes with a pro-proliferative, pro-survival phenotype and inflammatory changes akin to the effects of unopposed estrogen 27.

### The cooperative relationship of androgen (including testosterone and androstenedione) and insulin in type I EC

Apart from ovaries, estrogen could also be derived from the adrenal gland and peripheral fat conversion; accordingly, high fat levels (especially metabolic syndrome-related central obesity caused by hyperinsulinemia) are correlated with high peripheral fat and increased conversion of androgen to estrogen 17, 18. Further, excess insulin stimulates theca cell androgen activity, elevates serum-free testosterone levels via decreased hepatic SHBG production, amplifies luteinizing hormone (LH) and IGF-I-stimulated androgen production, and enhances serum IGF-I bioactivity via suppressed IGF binding protein production 28, 29. In the current study, we analyzed androgen in type I EC patients by multivariate logistic regression, adjusting for other positive characteristics (including insulin, estrone, and obesity factors) in a univariate analysis. According to these analyses, high testosterone was an independent risk factor of high estrone and insulin levels for type I EC (**Table 2**). High androstenedione is also associated with an increased risk of type I EC, but is not an independent risk factor (**Table 2**).

Epidemiological studies have shown that an increased risk of EC is associated with increased exposure to androgen, including variation in AR genes 30, 31 and PCOS, leading to hyperandrogenism. Hyperandrogenism is correlated to a high tolerance to insulin 32-34, which can lead to insulin level increases and other metabolic syndromes. Excessive androgen has been reported to alter autophosphorylation of the insulin receptor in the ovary of a woman with PCOS, resulting in a much higher tolerance to insulin 35. In addition, female rats exposed to androgen excess promote* Ins1* expression via transcriptional regulation that might contribute for basal hyperinsulinemia 33. These observations support the role of androgen in insulin tolerance; however, further studies are needed to clarify the causal basis.

On the other hand, apart from conversion into estrogen or the influence of insulin function, androgen itself normally regulates gene expression via AR, a positive prognostic indicator, and its loss is associated with shorter disease-free survival 21; therefore, the androgenic pathway as a therapeutic target for EC is not clearly established and requires further investigation. Our data revealed that the risk of both androgen and insulin increasing is higher than that of only a single factor increasing (Table 4), suggesting that androgen and insulin may have a synergistic effect on the occurrence of type I EC. Increasing evidence in prostate cancer and PCOS has revealed cross-talk between androgen and insulin signaling or its downstream cell proliferation pathways 36-38.

### The relationship between estrogen, androgen, and insulin

Our group has been exploring the role of sex hormones and growth factors in the development of EC. Importantly, by comparing the serum samples from patients with type 1 EC to the samples from healthy subjects, we discovered that high estrogen and insulin levels synergistically promoted cancer progression in the patients 39. Our clinical observations were confirmed by experiments in cell cultures and animal models 39. The underlying mechanism for producing the synergistic effect of estrogen and insulin levels in promoting type 1 EC progression was linked to the crosstalk between the hormones mediated by their cognate receptors 40.

Interestingly, using the serum samples from our previous study, we found that high serum androgen and insulin levels might be associated with a synergistic risk in the same patients 39. However, these results could not be directly confirmed by testing cognate hormone receptors, indicating that the underlying mechanism involves additional molecular components. Due to menstrual processes in women, the tissues producing the sex hormones differ in premenopausal and postmenopausal women. As discussed earlier, the ovary is the main producer for premenopausal estrogen, although other sites of estrogen biosynthesis are found throughout the body. These sites include the mesenchymal cells of adipose tissues and the skin, osteoblasts and perhaps chondrocytes in bones, vascular endothelial and aortic smooth muscle cells, and several sites in the brain 3. After menopause, circulating estrogen levels decline steeply, but testosterone levels alongside free testosterone levels did not vary by age, the mesenchymal cells of adipose tissues become the main estrogen producer due to the activity of aromatase that converts androgens to estrogens 3. Therefore, there is a correlation between estrogen and androgen levels.

Therefore, the question is whether androgens serve as a potential estrogen reservoir or if androgens interact with insulin via an unidentified signaling mechanism. Here, we hypothesize that a signaling mechanism is involved but we do not have enough evidence to support this hypothesis. Hence, the mechanism underlying the synergistic effect of androgen and insulin levels on the occurrence of type I EC should be examined in future studies.

In conclusion, our findings showed that high serum androgen levels and insulin concentrations have synergistic effects on type I EC risk, indicating that androgens and insulin could promote type I EC occurrence via different pathways. These findings provide a reference for the pathology of type I EC with respect to androgens and a basis for further studies of the precise mechanism underlying the effects of androgens.

## Figures and Tables

**Table 1:** Basic characteristics of the total, premenopausal and postmenopausal women with type I EC and healthy subjects

	Total women (n=1020)		*P* value	Premenopausal women (n=328)		*P* value	Postmenopausal women (n=692)		*P* value
	Cases (n=510)	Controls (n=510)		Cases (n=157)	Controls (n=171)		Cases (n=353)	Controls (n=339)	
Age	57.92±9.63	57.04±10.41	0.157	48.97±7.70	46.81±4.26	**0.002**	61.91±7.49	62.19±8.63	0.643
Height (cm)	159.69±5.52	159.86± 5.23	0.620	160.06±5.47	161.30±4.83	**0.031**	159.53±5.55	159.13±5.28	0.339
Weight (Kg)	69.47±13.50	62.98±9.57	**<0.001**	72.25±15.95	63.13±9.52	**<0.001**	68.25±12.08	62.91±9.61	**<0.001**
WC (cm)	91.36±10.87	85.90±10.88	**<0.001**	92.18±12.34	82.47±9.55	**<0.001**	91.00±10.15	87.633±11.11	**<0.001**
AC (cm)	97.52±11.76	88.99±10.73	**<0.001**	98.14±13.79	85.47±9.92	**<0.001**	97.25±10.74	90.77±10.70	**<0.001**
HC (cm)	99.80±11.35	97.72±7.48	**0.001**	100.56±13.69	97.49±7.24	**0.013**	99.74±8.65	97.83±7.61	**0.002**
BMI (Kg/m^2^)	27.15±4.99	24.65±3.62	**<0.001**	27.98±6.19	24.27±3.52	**<0.001**	26.78±4.32	24.85±3.66	**<0.001**
WHR	0.93±0.37	0.88±0.09	**0.004**	0.96±0.66	0.85±0.07	**0.022**	0.91±0.07	0.90±0.09	**0.006**
HOMA-IR	2.83(1.81-4.09)	1.76(1.18-2.63)	**<0.001**	3.51±3.93	1.95±1.20	**<0.001**	3.32±2.47	2.26±1.68	**<0.001**
Age at menopause	50.20±5.03	50.32±3.35	0.719	-	-	-	50.20±5.03	50.32±3.35	0.719
Nulliparous (%)	9.02	3.73	**0.001**	12.7	7.0	0.081	7.4	2.1	**0.001**
Irregular menstruation (%)	20.20	2.70	**<0.001**	49.7	7.8	**<0.001**	7.1	3.0	**0.045**
Diabetes (%)	28.04	6.27	**<0.001**	33.8	1.8	**<0.001**	25.5	8.6	**<0.001**
Hypertension (%)	47.45	22.94	**<0.001**	38.2	11.1	**<0.001**	51.6	28.9	**<0.001**
Family history of cancer (%)	29.41	16.27	**<0.001**	27.4	15.2	**0.007**	30.3	16.8	**<0.001**
Estrone (pg/mL)	46.76(33.08-65.04)	38.54(28.46-56.76)	**<0.001**	58.58(43.03-79.09)	58.480(39.29-84.18)	0.816	43.30(30.33-57.43)	34.52(25.59-43.66)	**<0.001**
Estradiol (pg/mL)	20.97(12.13-36.09)	21.01(14.62-34.73)	0.589	43.17(19.51-73.89)	63.29(22.26-104.82)	**0.032**	18.05(11.04-27.78)	18.60(12.00-23.77)	0.241
Insulin (μIU/mL)	10.37 (7.26-14.99)	7.53 (5.21-10.68)	**<0.001**	10.74(7.58-15.12)	7.53(5.22-10.24)	**<0.001**	10.13(7.12-15.00)	7.53(5.18-10.80)	**<0.001**
Testosterone (ng/dL)	43.20(33.00-59.00)	36.96(25.00-45.38)	**<0.001**	43.00(29.87-59.46)	35.70(21.73-46.00)	**<0.001**	43.40(31.73-58.72)	38.00(26.00-51.43)	**<0.001**
Androstenedione (ng/mL)	1.68 (1.16-2.40)	1.33 (0.92-31.94)	**<0.001**	1.86(1.30-2.53)	1.59(1.13-2.23)	**0.027**	1.63(1.12-2.34)	1.18(0.86-1.74)	**<0.001**
SHBG (nmol/L)	35.80(26.00-48.93)	48.80(34.95-66.60)	**<0.001**	34.20(24.50-48.00)	51.00(33.80-69.10)	**<0.001**	37.00(27.00-49.90)	47.80(36.00-66.10)	**<0.001**
FAI (%)	4.16(2.50-6.96)	2.53(1.50-4.20)	**<0.001**	4.25(2.45-7.62)	2.40(1.44-3.76)	**<0.001**	4.09(2.52-6.78)	2.65 (1.57-4.30)	**<0.001**
C-peptide (ng/mL)	1.30 (0.85-2.03)	0.95 (0.59-1.39)	**<0.001**	1.42(0.88-2.20)	0.88(0.54-1.23)	**<0.001**	1.27(0.84-1.90)	1.02(0.61-1.50)	**<0.001**

WC, waist circumference; AC, abdominal circumference; HC, hip circumference; BMI, body mass index; WHR, waist-to-hip ratio; HOMA-IR, homeostasis model assessment-insulin resistance; FAI, free androgen index. Continuous variables are shown as means (± standard deviation) whereas categorical variables are shown as their positive percentages; Medians (25-75%) of serum steroid sex hormones, insulin, C-peptide, and SHBG (sex hormone-binding globulin) concentrations in patients with endometrial cancer (EC) and control subjects; Bold text denotes statistical significance, *P*<0.05.

**Table 2:** Odds ratio for type I EC risk using logistic regression

	Univariate logistic regression			Multivariate logistic regression		
	OR	95% CI	*P* value	OR^a^	95% CI	*P* value
Testosterone (ng/dL)	1.656	1.293-2.121	**<0.001**	1.791	1.259-2.548	**0.001**
Androstenedione (ng/mL)	2.057	1.603-2.640	**<0.001**	0.959	0.658-1.397	0.826
SHBG (nmol/L)	0.335	0.260-0.432	**<0.001**	0. 475	0.331-0.683	**<0.001**
C-peptide (ng/mL)	2.504	1.946-3.222	**<0.001**	4.004	2.686-5.969	**<0.001**
Insulin (μIU/mL)	2.934	2.275-3.783	**<0.001**	0.845	0.564-1.265	0.414
Estrone (pg/mL)	2.041	1.590-2.619	**<0.001**	1.446	0.997-2.097	0.052
Estradiol (pg/mL)	1.000	0.782-1.278	1.000			
BMI (Kg/m2)	2.411	1.854-3.136	**<0.001**	0.983	0.663-1.457	0.932
WHR	3.894	2.871-5.282	**<0.001**	1.124	0.726-1.739	0.600
Diabetes (%)	5.820	3.875-8.741	**<0.001**	4.153	2.358-7.316	**<0.001**
Hypertension (%)	3.033	2.316-3.973	**<0.001**	2.155	1.466-3.166	**<0.001**
Nulliparous (%)	2.562	1.479-4.438	**0.001**	5.990	2.160-16.609	**0.001**
Irregular menstruation (%)	5.244	2.930-9.315	**<0.001**	3.301	1.753-6.218	**<0.001**
Family history of cancer (%)	2.144	1.584-2.901	**<0.001**	1.443	0.976-2.135	0.066

BMI, body mass index; WHR, waist-to-hip ratio; OR, odds ratio; CI, confidence interval. a: Adjusted for insulin, estrone, BMI, WHR, and family history of cancer. Bold text denotes statistical significance, *P*<0.05.

**Table 3:** The relationship between testosterone, androstenedione, insulin, FAI and type I EC risk after stratification for BMI, WHR, diabetes, hypertension using logistic regression model in total women

	Testosterone			Androstenedione			Insulin			FAI		
	OR	95% CI	*P* value	OR	95% CI	*P* value	OR	95% CI	*P* value	OR	95% CI	*P* value
**BMI**												
<24	1.202	0.787-1.837	0.394	1.626	1.062-2.489	**0.025**	2.748	1.706-4.427	**<0.001**	2.855	1.617-5.038	**<0.001**
≥24	1.885	1.375-2.585	**<0.001**	2.363	1.717-3.252	**<0.001**	2.304	1.660-3.196	**<0.001**	3.495	2.423-5.040	**<0.001**
**WHR**												
<0.85	0.694	0.402-1.200	0.191	1.699	0.991-2.914	0.054	2.921	1.621-5.265	**<0.001**	2.000	0.911-4.388	0.084
≥0.85	2.058	1.530-2.766	**<0.001**	2.097	1.559-2.820	**<0.001**	2.129	1.578-2.872	**<0.001**	3.298	2.341-4.645	**<0.001**
**Diabetes**												
No	1.722	1.308-2.266	**<0.001**	1.908	1.448-2.513	**<0.001**	2.581	1.951-3.414	**<0.001**	3.220	2.315-4.479	**<0.001**
Yes	1.500	0.693-3.246	0.303	2.830	1.282-6.249	**0.01**	1.851	0.823-4.165	0.137	5.035	1.836-13.811	**0.002**
**Hypertension**												
No	1.696	1.240-2.320	**0.001**	1.570	1.149-2.146	**0.005**	2.918	2.112-4.032	**<0.001**	3.959	2.718-5.766	**<0.001**
Yes	2.751	1.745-4.335	**<0.001**	1.686	1.081-2.630	**0.021**	1.683	1.059-2.675	**0.028**	3.006	1.755-5.147	**<0.001**

The cut-off points for serum testosterone, androstenedione and insulin concentrations were 40.10 ng/dL, 1.47 ng/mL and 8.82 mIU/mL, respectively. FAI ≥ 5%of serum levels were more than the reference interval limitation. OR, odds ratio; CI, confidence interval. Bold text denotes statistical significance, *P*<0.05.

**Table 4:** Serum higher androgen and insulin concentrations increase the risk for type I EC according to a logistic regression model

	Total women (n=1020)				Premenopausal women (n=328)				Postmenopausal women (n=692)			
	Casen=510 (%)	Controln=510 (%)	OR (95% CI)	*P* value	Casen=157 (%)	Controln=171 (%)	OR (95% CI)	*P* value	Casen=353 (%)	Controln=339 (%)	OR (95% CI)	*P* value
**Testosterone**												
<50%	223 (43.7)	287 (56.3)		Ref	66 (42.0)	98 (57.3)	Ref	Ref	160 (45.3)	189 (55.8)	Ref	Ref
≥50%	287 (56.3)	223 (43.7)	1.656 (1.293-2.121)	**<0.001**	91 (58.0)	73 (42.7)	1.851 (1.194-2.870)	**0.006**	193 (54.7)	150 (44.2)	1.520 (1.126-2.051)	**0.006**
**Androstenedione**												
<50%	210 (41.2)	301 (59.0)	Ref	Ref	72 (45.9)	92 (53.8)	Ref	Ref	135 (38.2)	214 (63.1)	Ref	Ref
≥50%	300 (58.2)	209 (41.0)	2.057 (1.603-2.640)	**<0.001**	85 (54.1)	79 (46.2)	1.375 (0.890-2.123)	0.151	218 (61.8)	125 (36.9)	2.765 (2.032-3.761)	**<0.001**
**Insulin**												
<50%	188 (36.9)	322 (63.1)	Ref	Ref	50 (31.8)	114 (66.7)	Ref	Ref	139 (39.4)	207 (61.1)	Ref	Ref
≥50%	322 (63.1)	188 (36.9)	2.934 (2.275-3.783)	**<0.001**	107 (68.2)	57 (33.3)	4.280 (2.695-6.796)	**<0.001**	214 (60.6)	132 (38.9)	2.414 (1.779-3.277)	**<0.001**
**Testosterone+Insulin**												
***Testosterone<50%***												
Insulin												
<50%	101 (19.8)	179 (35.1)	Ref	Ref	22 (14.0)	63 (36.8)	Ref	Ref	78 (22.1)	113 (33.3)	Ref	Ref
≥50%	122 (23.9)	108 (21.2)	2.002 (1.403-2.857)	**<0.001**	44 (28.0)	35 (20.5)	3.600 (1.865-6.950)	**<0.001**	82 (23.2)	76 (22.4)	1.563 (1.022-2.391)	**0.039**
***Testosterone ≥50%***												
Insulin												
<50%	87 (17.1)	143 (28.0)	1.078 (0.751-1.547)	0.683	28 (17.8)	51 (29.8)	1.572 (0.805-3.071)	0.185	61 (17.3)	94 (27.7)	0.94 (0.610-1.449)	0.78
≥50%	200 (39.2)	80 (15.7)	4.431 (3.104-6.325)	**<0.001**	63 (40.1)	22 (12.9)	8.200 (4.128-16.291)	**<0.001**	132 (37.4)	56 (16.5)	3.415 (2.232-5.225)	**<0.001**
**Androstenedione+Insulin**												
***Androstenedione<50%***												
Insulin												
<50%	92 (18.0)	195 (38.2)	Ref	Ref	25 (15.9)	67 (39.2)	Ref	Ref	63 (17.8)	137 (40.4)	Ref	Ref
≥50%	118 (23.1)	106 (20.8)	2.360 (1.645-3.385)	**< 0.001**	47 (29.9)	25 (14.6)	5.038 (2.583-9.828)	**<0.001**	72 (20.4)	77 (22.7)	2.033 (1.312-3.152)	**0.002**
***Androstenedione***≥5***0%***												
Insulin												
<50%	96 (18.8)	127 (24.9)	1.602 (1.115-2.303)	**0.011**	25 (15.9)	47 (27.5)	1.426 (0.731-2.781)	0.298	76 (21.5)	70 (20.6)	2.361 (1.519-3.669)	**<0.001**
≥50%	204 (40.0)	82 (16.1)	5.273 (3.692-7.532)	**<0.001**	60 (38.2)	32 (18.7)	5.025 (2.680-9.421)	**<0.001**	142 (40.2)	55 (16.2)	5.614 (3.648-8.641)	**0.001**
**FAI+Insulin**												
***FAI <5%***												
Insulin												
<50%	137 (26.9)	286 (56.1)	Ref	Ref	36 (22.9)	106 (62.0)	Ref	Ref	103 (29.2)	179 (52.8)	Ref	Ref
≥50%	172 (33.7)	148 (29.0)	2.426 (1.798-3.274)	**<0.001**	54 (34.4)	48 (28.1)	3.312 (1.926-5.698)	**<0.001**	116 (32.8)	101 (29.8)	1.996 (1.392-2.862)	**<0.001**
***FAI***≥5***%***												
Insulin												
<50%	51 (10.0)	36 (7.1)	2.957 (1.843-4.745)	**<0.001**	14 (8.9)	8 (4.7)	5.153 (1.998-13.289)	**0.001**	36 (10.2)	28 (8.3)	2.234 (1.289-3.873)	**0.004**
≥50%	150 (29.4)	40 (7.8)	7.828 (5.227-11.724)	**<0.001**	53 (33.8)	9 (5.3)	17.340 (7.780-38.644)	**<0.001**	98 (27.8)	31 (9.1)	5.494 (3.430-8.799)	**<0.001**

The cut-off points for serum androstenedione concentrations were 1.47, 1.75 and 1.39 ng/mL in total, premenopausal and postmenopausal women, respectively; The cut-off points for serum testosterone concentrations were 40.10, 39.02 and 41.00 ng/dL in total, premenopausal and postmenopausal women, respectively; The cut-off points for serum insulin concentrations were 8.82, 8.74 and 8.90 mIU/mL in total, premenopausal and postmenopausal women, respectively; FAI ≥5% of serum levels were more than the reference interval limitation; OR, odds ratio; CI, confidence interval; Bold text denotes statistical significance, *P*<0.05.
